# Fungal diversity and community structure from coastal and barrier island beaches in the United States Gulf of Mexico

**DOI:** 10.1038/s41598-021-81688-5

**Published:** 2021-02-16

**Authors:** Allison K. Walker, Brent M. Robicheau

**Affiliations:** 1 Department of Biology, Acadia University, Wolfville, NS B4P 2R6 Canada; 2 Department of Biology, Dalhousie University, Halifax, NS B3H 4R2 Canada

**Keywords:** Microbial communities, Fungi, Marine biology

## Abstract

Fungi are an important and understudied component of coastal biomes including sand beaches. Basic biogeographic diversity data are lacking for marine fungi in most parts of the world, despite their important role in decomposition. We examined intertidal fungal communities at several United States (US) Gulf of Mexico sand beach sites using morphology and *ITS* rDNA terminal restriction fragment length polymorphism (T-RFLP) analyses. Fungal biogeographical patterns from sand beach detritus (wood, emergent plant [mangrove/ saltmarsh], or marine [algae, seagrass]) from Florida, Mississippi, and Texas were investigated using diversity indices and multivariate analyses. Fungal diversity increased with decreasing latitude at our study sites. Substrate type strongly influenced fungal community structure in this region, with different fungal communities on detrital marine versus emergent substrates, as well as detrital marine versus wood substrates. Thirty-five fungi were identified morphologically, including new regional and host records. Of these, 86% were unique to an individual collection (i.e., sampled once from one site). Rarefaction curves from pooled morphological data from all sites estimate the number of samples required to characterize the mycota of each substrate. As sampling occurred before the Deepwater Horizon oil spill (April-2010), our findings contribute pre-oil spill sand beach biodiversity data and marine fungal distribution trends within this economically important oceanographic region.

## Introduction

Fungi are an important but often overlooked component of coastal ecosystems. An estimated > 10,000 marine fungal species exist globally^1,2^, of which only 1,000 have been described^3,4^. Primarily saprotrophic, fungi are vital to coastal nutrient cycling processes and food webs^1^; however, basic distribution data for coastal fungi are lacking in many parts of the world, as is knowledge of the sampling intensity required to characterize this underexplored component of marine biodiversity. Progress has been made in the inventorying of intertidal beach fungi. Danish studies by Koch (1974), documented 37 fungi on manufactured wood (driftwood) on the northwest coast of Jutland with 5 *Corollospora* species dominating the sand beach-dune interface zone^5^, as well as Rees et al. (1979) who documented over 50 species^6^, and Rees and Jones^7^ who documented 47 species on 9 nutrient sources. More recently, 1–2 beaches in each of Egypt^8^, Portugal^9,10^, the South Baltic Sea^11^, Malaysia and Singapore^12^, Italy^13^, and a larger intertidal area in Norway^14^ have also been investigated. Species of filamentous higher marine fungi have been summarized^3,15,16^ with clear indication that many fungi identified from marine environments belong to the phylum Ascomycota^4^. Fungi are known from coastal, open-ocean and deep-sea waters, and colonize a variety of substrates including detrital wood, algae, plants and animals^1,2,17^. Due to their ability to degrade complex substrates such as lignocellulose, keratin, chitin and calcareous structures, fungi are important decomposers of plant and animal-based marine detritus^18–20^. Ascomycete fungi are the principle decomposers of the dominant Gulf of Mexico emergent saltmarsh plants *Spartina alterniflora* (Fam. Poaceae) and *Juncus roemerianus* (Fam. Juncaceae)^21,22^, and marine ascomycetes are also known as symbionts and pathogens of marine algae and marine fauna^19,20^. Prior to our work, ~ 251 ascomycete genera containing ~ 424 species were known from marine environments globally^23^, with 21 ascomycete genera and 45 species known from the Gulf of Mexico^15,24^. The excellent curated website, www.marinefungi.org, should be consulted for up-to-date values on the accepted number of marine fungal taxa (webpage last accessed Jul-30–2020)^16^.

The Gulf of Mexico is the ninth largest body of water in the world and North America's most economically productive ecosystem^25^. The U.S. Gulf of Mexico spans 2,703 km of coastline and receives freshwater and detritus from 33 major rivers draining from 31 U.S. states^26^. This biogeographic zone is warm-temperate to subtropical, and is of special interest mycologically because subtropical zones are predicted to contain a high diversity of cosmopolitan, temperate, sub-tropical and tropical marine fungal species^27^. The warm-temperate North Gulf region invites study of the role of season on coastal fungal communities.

**Figure 1:**
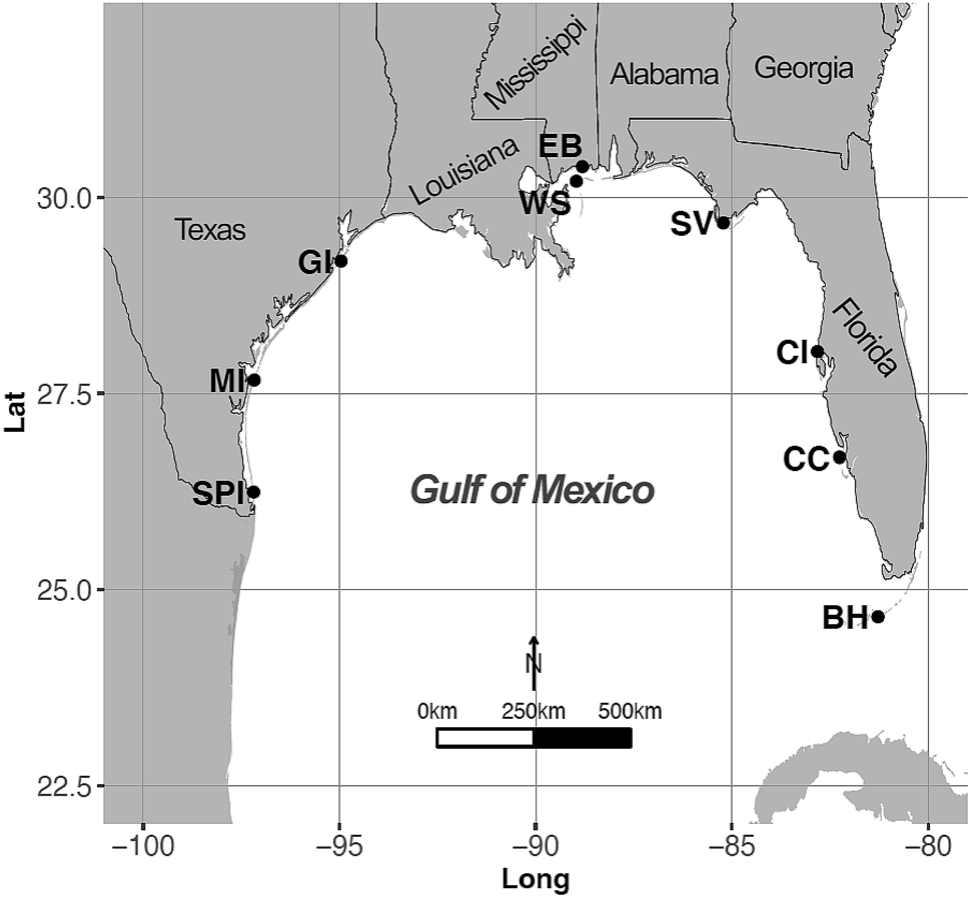
Sand beach collection sites along the U.S. Gulf of Mexico coastline used to study fungal diversity.

We characterized fungal diversity and distribution patterns at several sites in the U.S. Gulf of Mexico by collecting fungi from intertidal substrates along latitudinal gradients at barrier island sand beach sites in Texas, Mississippi, and Florida (Fig. 1). Our two objectives were: (i) to morphologically characterize fungal communities occurring on major intertidal substrate types (sand, seafoam, marine plant detritus, emergent plant detritus, and wood) using direct microscopy, and (ii) to use *ITS* rDNA T-RFLP analyses (a community molecular detection method) to obtain species occurrence and relative abundance data to assess the roles of latitude (warm-temperate versus subtropical), season (summer versus winter), substrate type (wood, marine plant detritus, emergent plant detritus), and environmental factors (salinity, water temperature, pH) in structuring the fungal communities observed. We provide a comparison of fungal diversity across several sites along the coastal United States (US) Gulf of Mexico focusing on multiple substrates using both microscopy and community molecular detection methods. We include multivariate statistical analysis of the environmental factors shaping fungal distribution at our study sites. Our work further complements the southern Gulf of Mexico sand beach fungal inventory by Velez et al.^24^, as well as earlier marine mycological research in this region^28^.

## Results

### Morphological analyses

**Figure 2:**
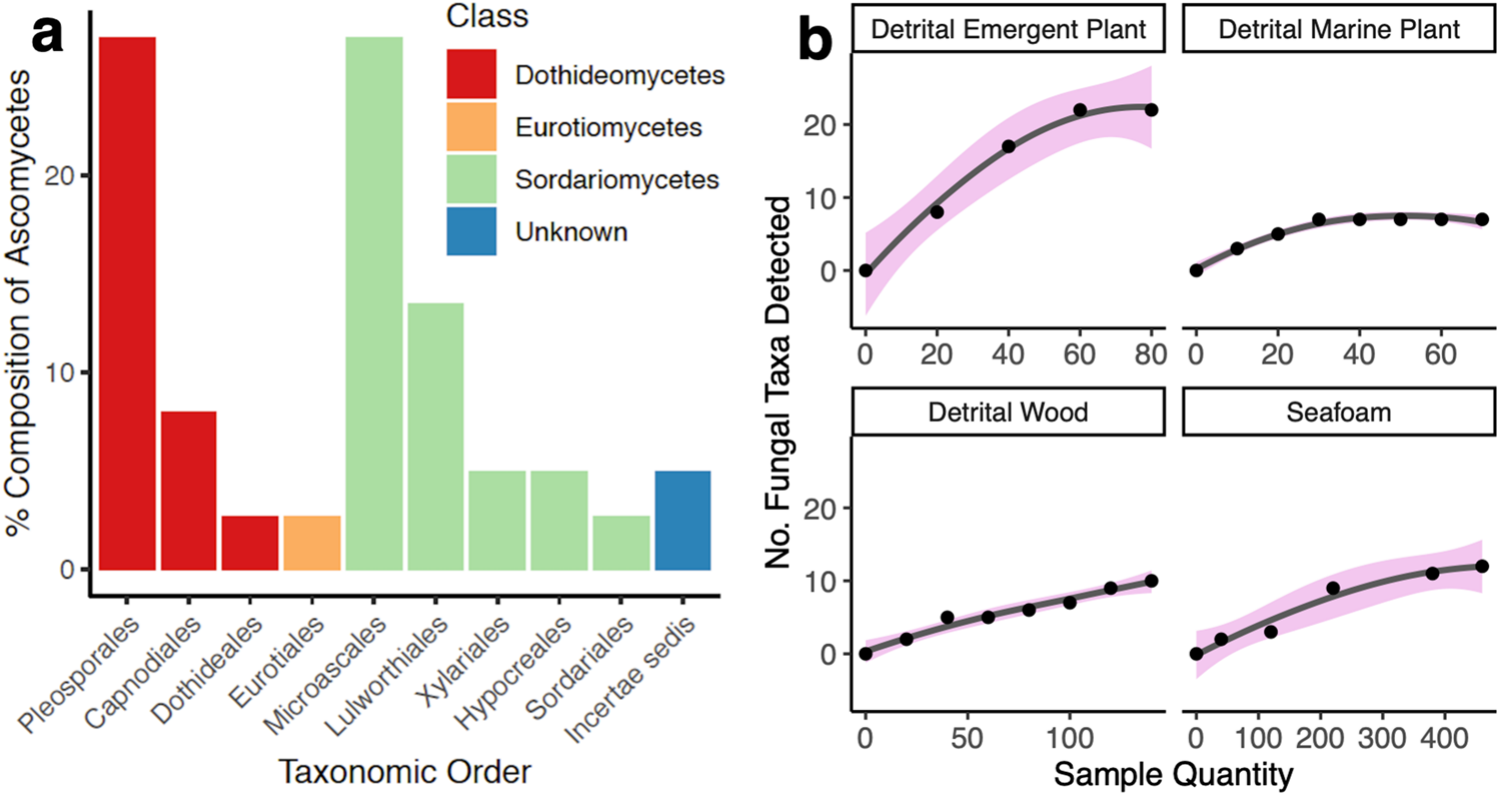
(**a**) Taxonomic composition of ascomycetes identified morphologically during this study (expressed as a percentage of the total [288]). (**b**) Relationship between the number of taxa detected morphologically and the number of samples collected for each sample type.

**Figure 3:**
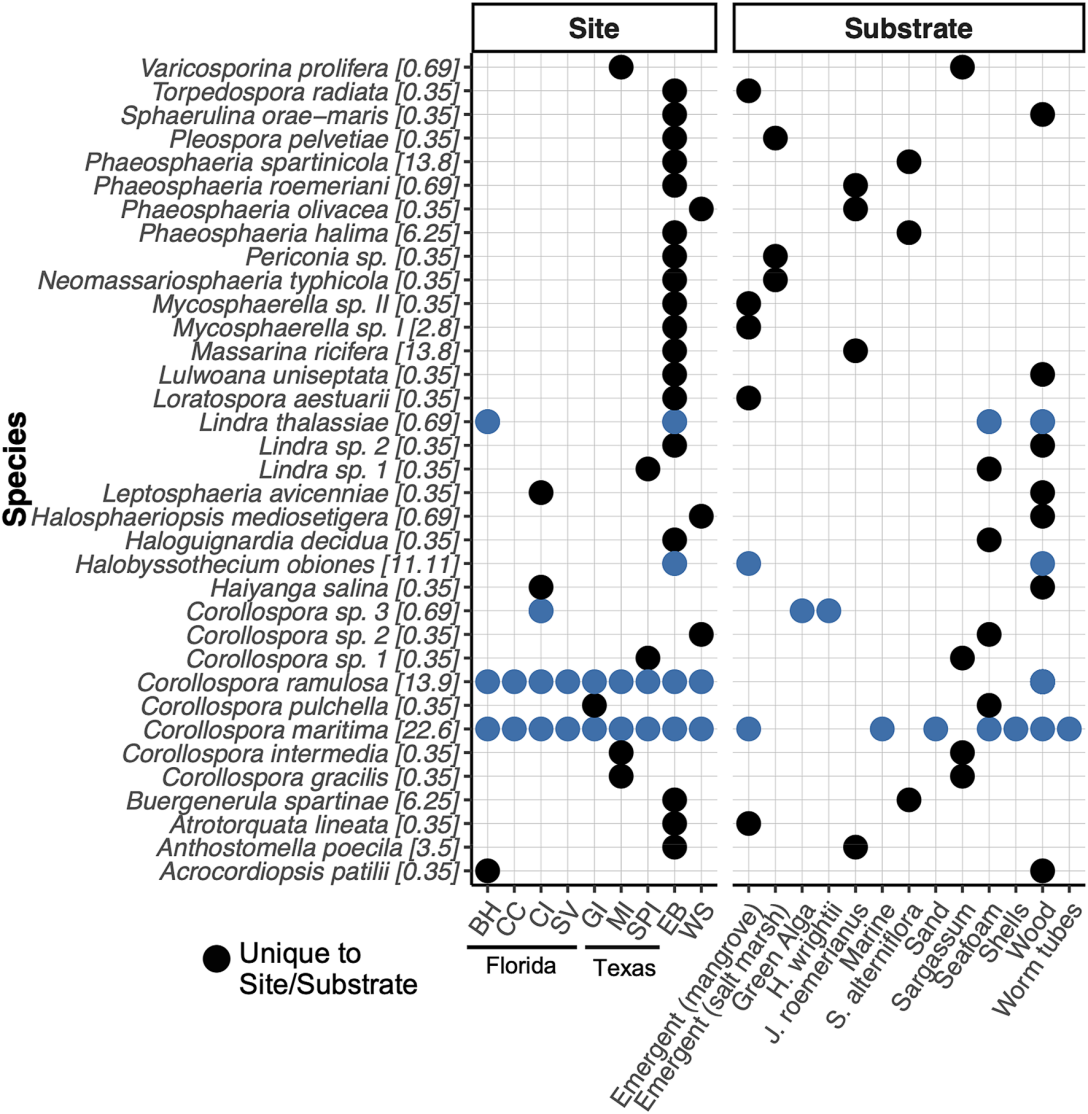
Overview of fungi detected morphologically on intertidal substrates of the U.S. Gulf of Mexico by site (left panel) and substrate-type (right panel). Substrate type ‘marine’ includes unidentified seagrass and algae. Substrate type ‘Emergent (salt marsh)’ includes salt marsh plant detritus not identifiable to host plant species. The relative frequency of occurrence for fungal taxa in all collections housing sporulating fungi (*n* = 288) as detected morphologically is provided in brackets next to taxon labels. For location and further description of sites see Fig. 1 and Supplementary Table S4. Collections occurred weekly at East Beach, Mississippi (EB); collections occurred bimonthly at West Ship Island, Mississippi (WS) and once per winter and per summer for the remainder of sites. Taxonomic authorities are provided in Supplementary Table S1.

Seven hundred and fifty collections of beach detritus, sand, and seafoam were made from the U.S. Gulf of Mexico, with 288 of these collections (38.4%) supporting sporulating fungi (35 different ascomycetes). Some fungi observed from intertidal substrates were morphologically unidentifiable to species level. Of those identified to genus, 30% were anamorphic (asexual) ascomycetes and 70% were sexual ascomycetes. The taxonomic composition of the U.S. Gulf of Mexico fungal assemblage based on our morphological data is shown in Fig. 2a. Of the fungi identified, 86% were unique to an individual collection (i.e., in one sample from one site). Frequency of occurrence of each marine ascomycete that could be identified to genus, along with substrate presence/absence information is given in Fig. 3 (see values in square brackets within Fig. 3 for frequency of occurrence).

To provide further context, fungal species identifications reported from the Gulf of Mexico after our sampling had occurred, including those from marine sediment, water, seagrass, and (or) detritus are given in Supplementary Table S2. Of the fungi identified to species level (from Fig. 3), only 7 have been noted in studies conducted after our sampling took place. Nineteen fungal taxa were documented in the sand beach inventory of the southern Gulf of Mexico by Velez et al.^24^, who noted many of the same genera as our study (e.g. *Corollospora*, *Lindra*, *Mycosphaerella*). Although we focused primarily on underdeveloped sites along the U.S. (Northern) Gulf of Mexico coast (to decrease the effect of human disturbance), it is important to note that many of the genera, as well as several species that we identified overlap with previous studies from the state of Florida that were summarized by Jones and Puglisi^28^. Of the names listed in Fig. 3, the following species were also listed by Jones and Puglisi^28^: *Buergenerula spartinae*, *Corollospora maritima*, *Lindra thalassiae*, *Phaeosphaeria halima*, *Torpedospora radiata*, *Trichocladium achrasporum (*= *Halosphaeriopsis mediosetigera)*, and *Variocosporina ramulosa (*= *Corollospora ramulosa*). Furthermore, the following genera identified in our study were also documented by Jones and Puglisi^28^ from the state of Florida: *Anthostomella*, *Leptosphaeria*, *Massarina*, *Mycosphaerella*, *Passeriniella*, *Periconia*, *Pleospora*, and *Zalerion*.

Rarefaction (sampling) curves generated from morphological data from all eight sites estimate the number of samples required to characterize the mycota of each substrate type (Fig. 2b). The sampling curve was not saturated for seafoam, and in 460 collections of seafoam only 12 fungal taxa were detected through microscopic examination of spores (Fig. 2b). Conversely, the sampling curve appears saturated around 70 samples for detrital emergent plant substrate (saltmarsh/mangrove) (Fig. 2b), and around 30 samples for detrital marine plant/algal substrate (seagrass/algae) (Fig. 2b). The accumulation of species found on wood slowed at around 130 samples, indicating approximately this many samples may be required to characterize the fungi from this substrate type using morphological methods (Fig. 2b). Emergent plant (saltmarsh) detritus had high fungal diversity as detected through this approach; perhaps due to this substrate’s high lignocellulose content.

### Community characterization (via *ITS* T-RFLP)

#### Seasons and substrates

When all Gulf of Mexico samples were analyzed for the effect of season on community composition using a one-way ANOSIM, there was no statistically significant seasonal effect for the US Gulf of Mexico on ascomycete community species richness or relative abundance on any substrate type (R = 0.03, *P* > 0.1). However, cluster and NMDS analyses did show slight seasonal effects within a substrate. For example, ascomycete communities on intertidal wood from South Padre Island, Texas were distinct in summer and winter (Supplementary Fig. S1a). When winter collections were examined, wood, emergent (saltmarsh) and marine plant detritus communities from Mustang and South Padre Islands grouped together. Less similarity was seen in summer wood fungal communities from South Padre Island and Mustang Island, although they did group together (Supplementary Fig. S1a). Summer and winter detrital saltmarsh communities from Galveston Island showed only 10% similarity (Supplementary Fig. S1a); seasonality may structure saltmarsh fungal communities. Similar fungal species richness was documented for wood and emergent plant detritus in both summer and winter. Within all three substrate types, samples separated out in the NMDS analysis based on season (Supplementary Fig. S1b). Of note, marine detritus had higher species richness in winter, with up to 31 fungi detected from a single sample.

Our morphological inventory detected distinct fungal communities on distinct substrate types, with some overlap of abundant generalist species such as *Corollospora maritima* and *Halobyssothecium obiones*. Although our T-RFLP results indicated similar fungal species richness for wood and emergent detritus, our morphological inventory revealed more species fruiting on emergent detritus (saltmarsh) at the time of collection than for other substrate types. This high diversity may represent functional redundancy of saltmarsh fungal taxa in the presence of large quantities of lignocellulose. Indeed, the highest species richness was encountered on substrates rich in lignocellulose (for e.g., in summer samples: for wood the mean number of spp. = 15 and for emergent plant detritus the mean number of spp. = 14, versus marine plant detritus where the mean number of spp. = 6; values standardized to sampling effort). Marine plant/algal detritus, which contains less lignocellulose, exhibited the lowest species richness compared to wood and emergent plant detritus for both seasons (summer and winter).

T-RFLP community data were only collected for one substrate at St. Vincent Island in FL (summer *Sargassum*) and it shared < 30% species in common with Cayo Costa and Caladesi Island, FL marine substrates. All substrate pairs differed significantly (*P* < 0.05) in species composition with the exception of the Emergent/Wood substrate pair (*P* > 0.1; Table 1).

**Table 1:** Global R statistic and pairwise comparisons of fungal community similarities between different substrates based on T-RF species relative abundance data.

Pair of Substrates	Global R Statistic	Significance	No. of observations
	0.247	*P* < 0.02	28
	R statistic	Significance	No. of observations
Marine/Emergent	0.335	**P < 0.02**	11
Marine/Wood	0.226	**P < 0.05**	9
Emergent/Wood	− 0.075	P > 0.1	337

R statistics and *p*-values from ANOSIM. Bold font shows statistically significant values.

We also noted differences in ascomycete communities within substrate type, for example marine detritus at Mustang Island in Texas. The two seagrass species, and one green algal species examined, shared ~ 18% ascomycete species (8/45 species), with each host exhibiting a different dominant ascomycete species based on T-RFLP percent abundance data. The seagrass *Thalassia testudinum* contained the most diverse ascomycete community, with all three samples examined exhibiting similar species richness (22, 18, and 18 species, respectively).

#### Latitude

**Figure 4:**
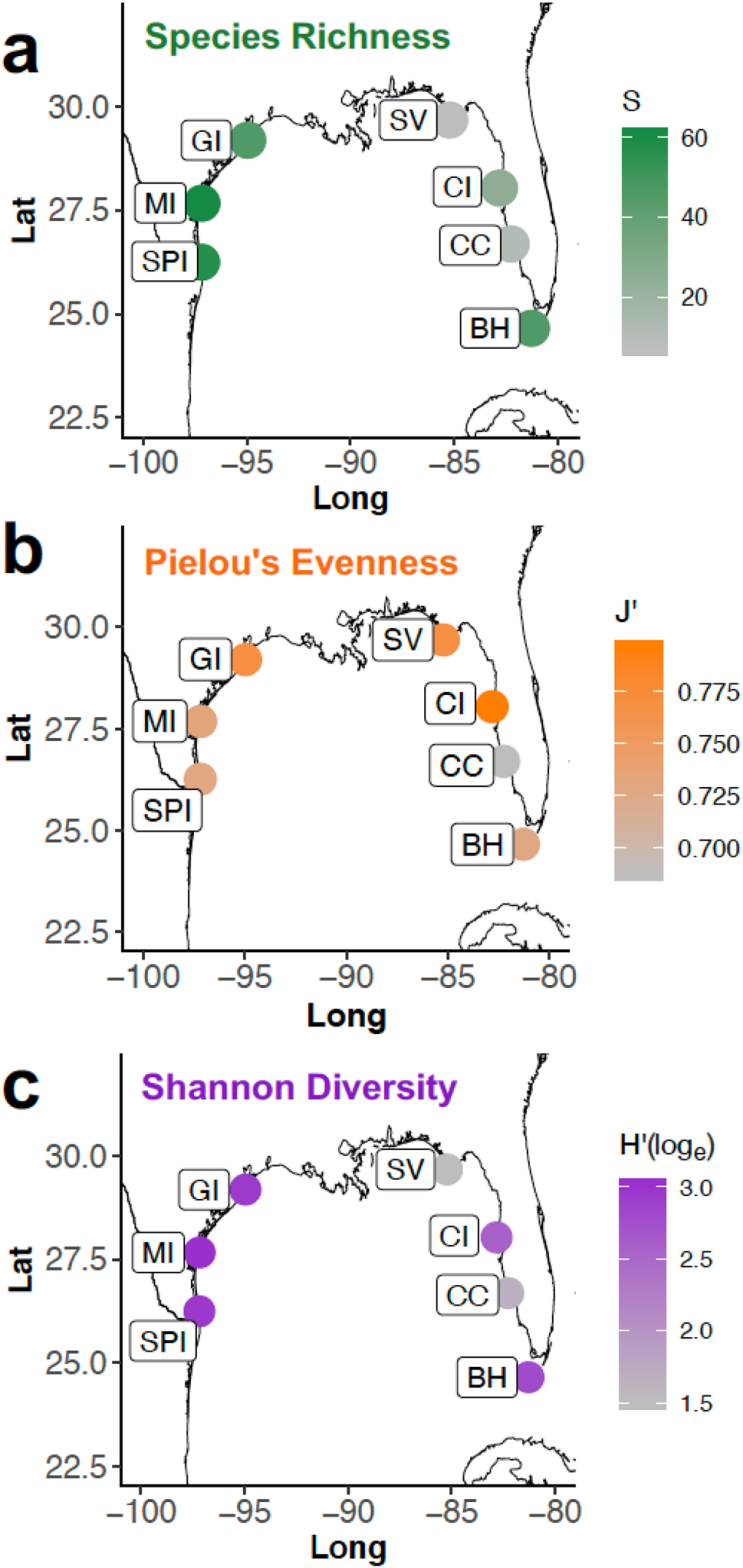
(**a**) Species richness, (**b**) Pielou’s evenness and (**c**) Shannon diversity by site as calculated from T-RFLP species richness and relative abundance data. Data used to generate figure are provided in Supplementary Table S3.

We observed latitudinal differences in fungal communities colonizing the same substrate type. Fungal species richness increased with decreasing latitude in both Texas and Florida, when all substrate types were pooled. Higher species richness was noted on detrital seagrass at lower latitudes in Florida than at the Florida Panhandle site examined (SV). Alpha diversity (species richness; Fig. 4) was highest at Site SPI [species richness (S) = 57] and Site MI [S = 61] in Texas, and at Site BH [S = 46], the southernmost site sampled in Florida, based on T-RFLP data.

**Figure 5:**
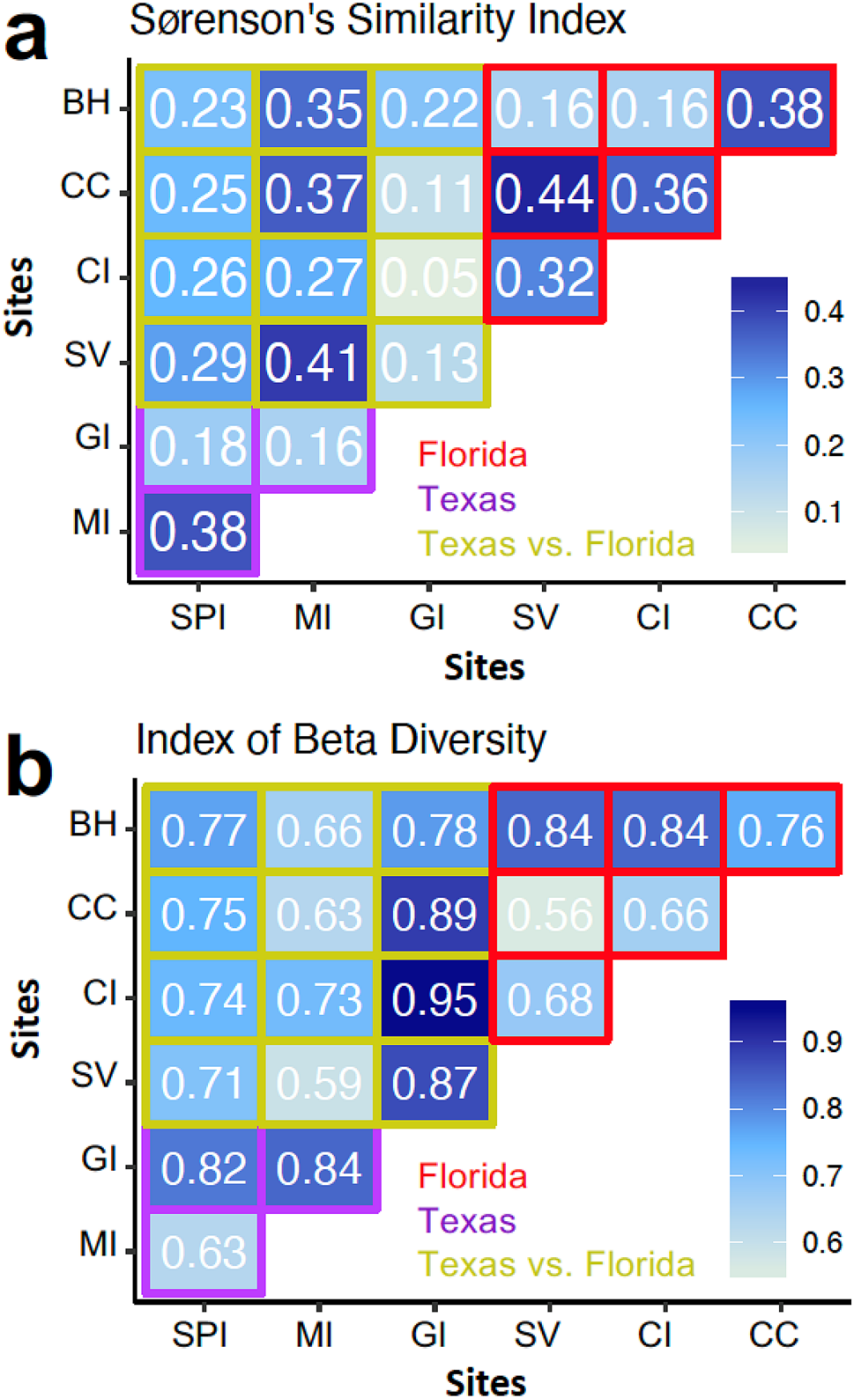
(**a**) Sørenson’s similarity index and (**b**) index of beta diversity^29^ for Texas and Florida collection sites based on *ITS* rDNA T-RFLP data.

**Figure 6:**
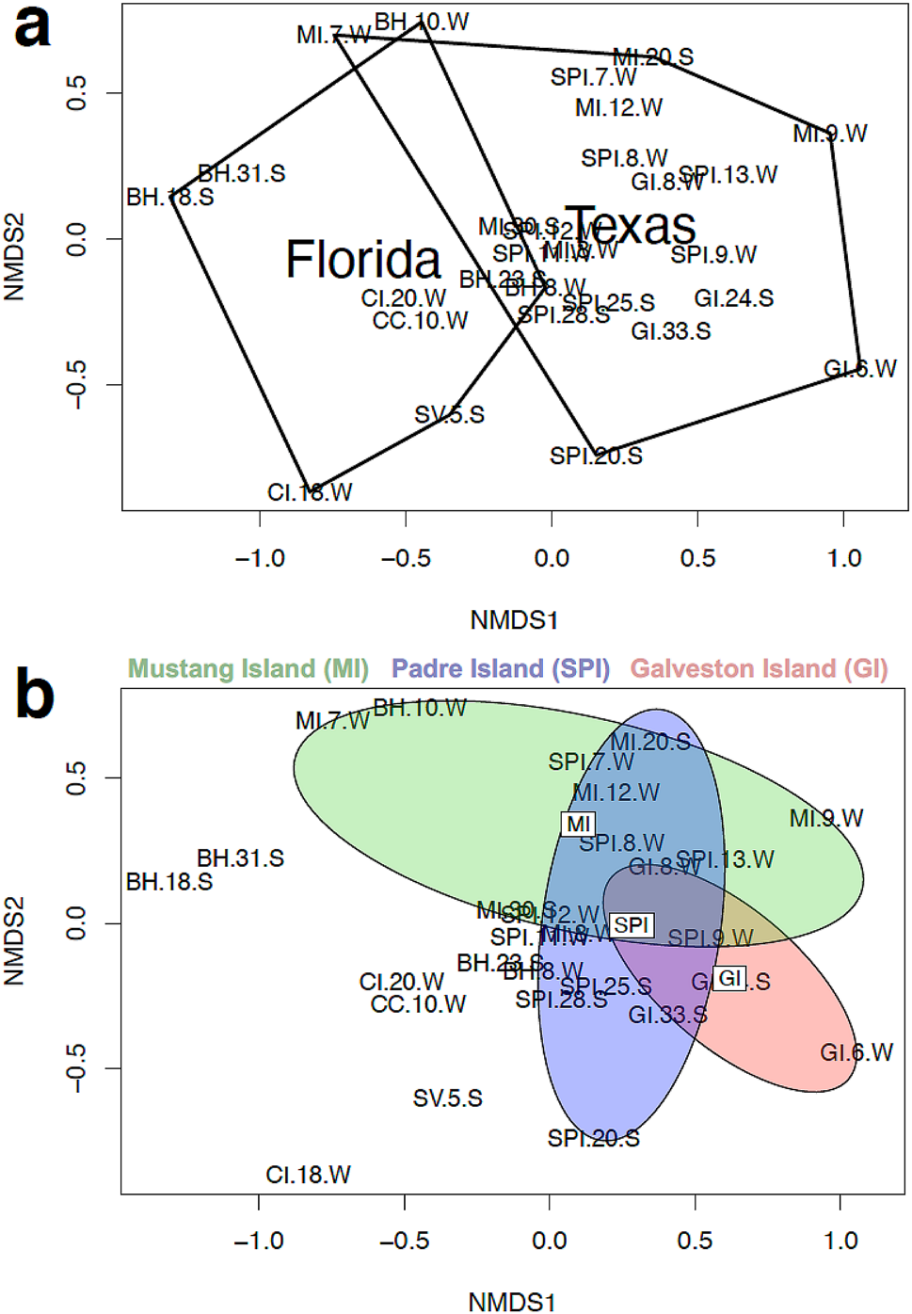
Gulf of Mexico *ITS* T-RF relative species abundance data visualized by 2D NMDS ordination using a Bray–Curtis similarity matrix. (**a**) Data points are enclosed based on whether they are from Florida or Texas; a convex hull circles all points within Florida or within Texas^30^. (**b**) Data points are enclosed based on their location within Texas; an ellipsoid hull^30^ groups data within MI, SPI, or GI. Refer to Supplementary Table S4 for location names, data were *log b* (*x*) + 1 transformed for *x* > 0 [where *b* = base of the logarithm]^31^.

Beta diversity (amount of species change between sites) was highest between Site GI in Texas and Site CI in Florida (Fig. 5). Beta diversity was lowest between Site SV in Florida and Site CC in Florida (44% similarity; Fig. 5). Surprisingly, in terms of fungal diversity, the most similar sites based on Sørenson’s Index were Site SV in Florida and Site MI in Texas (41% similarity; Fig. 5). However, the southernmost sites in both Texas and Florida had similar beta diversity (Site MI and Site SPI (38%) in Texas; and Site CC and Site BH in Florida (38%); Fig. 5). Little overlap was found between Texas and Florida ascomycete communities when visualized by NMDS, other than for the southernmost sites (South Padre Island “SPI” in Texas and Bahia Honda “BH” in Florida) (Fig. 6a). For Texas samples, cluster and NMDS visualization revealed fungal communities from South Padre Island (SPI) and Mustang Island (MI) intertidal substrates clustered together while most fungal communities from Galveston Island (GI) were distinct (Fig. 6b and Supplementary Fig. S1a). See Supplementary Fig. S2 for a Shepard diagram of how the stress of the NMDS was calculated. For site comparisons, significant differences (*p*-value < 0.05) in ascomycete communities were noted between four site pairs using analysis of similarities (ANOSIM) (Table 2).

**Table 2:** Pairwise comparisons of fungal community similarities between different sites based on T-RF species relative abundance data for all substrates.

Pair of sites	Global R Statistic	Significance	No. of observations
	0.322	*P* < 0.004	28
	R Statistic	Significance	No. of observations
BH, CC	0.4	*P* > 0.1	1
BH, CI	0.491	***P < 0.05***	1
BH, GI	0.494	***P = 0.04***	5
BH, MI	0.251	*P* < 0.07	32
BH, SPI	0.495	***P = 0.004***	3
BH, SV	0.4	*P* > 0.1	1
CC, CI	0	*P* > 0.1	2
CC, GI	0.25	*P* > 0.1	2
CC, MI	− 0.067	*P* > 0.1	5
CC, SPI	0.395	*P* > 0.1	3
CI, GI	0.679	*P* < 0.07	1
CI, MI	0.229	*P* > 0.1	6
CI, SPI	0.637	*P* < 0.06	3
CI, SV	0	*P* > 0.1	2
GI, MI	0.095	*P* > 0.1	50
GI, SPI	0.343	***P < 0.05***	32
GI, SV	0.833	*P* > 0.1	1
MI, SPI	0.028	*P* > 0.1	347
MI, SV	0.356	*P* > 0.1	3
SPI, SV	0.66	*P* = 0.1	1

R statistics and *p*-values from ANOSIM. Bold font shows statistically significant *P*-values < 0.05.

## Discussion

Our study documented similar species to the Florida study of Jones and Puglisi^28^ (7 species and 8 genera in common). However, several fungi we detected deviated from published descriptions or represented new records for the substrate or region. For instance, we detected three morphotypes of *Corollospora* from four different substrates (*Sargassum* sp., seafoam, *Halodule wrightii,* and a green alga) at three collection sites: South Padre Island, West Ship Island, and Caladesi Island. These three *Corollospora* species (with differing morphologies) did not match any published *Corollospora* species descriptions (an assertion based on ascospore and ascospore appendage morphology). A new species of *Corollospora* from calcareous material associated with the seagrass *Zostera marina* and another from the shell of a shipworm in decayed driftwood (both from Egypt)^32^ along with more recent work by Tibell^33^ and Réblová et al.^34^ suggest additional species may exist. This adds to work by Nakagiri and Tokura^35^ describing seven new *Corollospora* species from sand beaches of Japan. Recent literature points to 29 described species of *Corollospora*^3^, however the curated online resource marinefungi.org should be consulted for the most up-to-date number of *Corollospora* species^16^. Additionally, two morphospecies of *Lindra* were detected in our study, one from seafoam at South Padre Island in Texas and one from detrital wood at East Beach in Mississippi, both with shorter ascospores than previously documented for the genus *Lindra*. Further research is required to determine if the unique *Lindra* and *Corollospora* morphologies observed represent uncharacterized species.

There were two other species occurrences of note at our study sites. The first is *Phaeosphaeria olivacea*, first described from *Juncus roemerianus* in North Carolina (Atlantic coast)^36^. Here we report *P. olivacea* for the first time from the Gulf of Mexico at West Ship Island, Mississippi, on intertidal detrital *J. roemerianus*. The second occurrence of note was *Acrocordiopsis patilii* on detrital wood at Bahia Honda, Florida. This fungus was previously reported from mangrove wood from India and Brunei^37^; here we report it for the first time from the United States.

The fungi identified morphologically primarily belonged to the classes Sordariomycetes and Dothideomycetes (Phylum Ascomycota), which represent a wide range of ecologies including pathogens and endophytes of plants, animal pathogens, mycoparasites and lichenized fungi^38^. The following ascomycetes (found as ascomata on detrital wood) were previously reported from mangrove wood: *Acrocordiopsis patilii* at Bahia Honda in Florida, as well as *Haiyanga salina* and *Leptosphaeria avicenniae* at Caladesi Island in Florida. The anamorphic ascomycete *Varicosporina ramulosa* (now *Corollospora ramulosa*) was also common on wood throughout our Gulf of Mexico study sites. Other fungi we documented from intertidal substrates may have terrestrial origins. *Cochliobolus hawaiiensis* is a ubiquitous plant pathogen of rice, maize, sorghum, millet and sugar cane^39^. *Acremonium alternatum* is a hyaline hyphomycete and a saprobic, opportunistic mammalian pathogen, as well as plant endophyte (fungus occurring inside asymptomatic plant tissue) and entomopathogen^40^. Furthermore, *Alternaria tenuissima* is a cosmopolitan Pleosporalean plant pathogen and is the causal agent of leaf spot of eggplant^41^. Rarely pathogenic to humans, wind dispersed *Cladosporium* spores are found in abundance globally. For example, one species, *Cladosporium carrionii*, is a causal agent of chromoblastomycosis in subtropical and tropical regions^42,43^. The identification of novel *Cladosporium* continues to be of interest. For instance, an unidentified marine-derived species of *Cladosporium* was found to produce antibiotic and antifouling compounds in culture, thereby inhibiting attachment of bryozoan larvae and also adversely affecting the growth of six bacterial species^44^. *Paecilomyces variotii* is a hyaline hyphomycete common in air and food, but it is also associated with many types of human infections and is among the emerging causative agents of opportunistic mycoses in immunocompromised hosts, causing hyalohyphomycosis^45^. The species listed suggest that terrestrial inputs are likely influencing the fungal diversity we documented at our study sites^46^.

*Corollospora maritima* was the most frequently observed arenicolous species, and in combination with *Corollospora ramulosa,* were the most common ascomycete species on solid substrates, present at all sites in both seasons. In particular, several of these solid substrates were physically connected (i.e., sand, seafoam, wood, and shells). *Corollospora maritima* colonized sand grains atop other intertidal substrates, such as wood, shells, worm tubes and *Sargassum*, with the ascomata developing on the sand grains and hyphae extending into the other substrates, thus creating an intersection between sand grains and other marine substrate types. As *Corollospora* species possess tough carbonaceous ascomata resistant to desiccation, attachment to sand grains may facilitate the persistence and ubiquity of *Corollospora* species in the dynamic intertidal zone of Gulf of Mexico sand beaches. Furthermore, another species of *Corollospora, C. ramulosa,* forms sclerocarps similar to ascomata of *Corollospora* spp. to aid in its persistence in the intertidal zone^47^. It is important to note that visible organic matter was not observed within the sand at the sites sampled and arenicolous fungal diversity was lower than expected when compared with studies of other sandy beaches in Mexico, Cuba, Texas, Japan and elsewhere^35,48–53^. Freshwater inputs from the San Jacinto River (Galveston TX site) and Mississippi River (Mississippi sites) contribute to lower salinities in these coastal waters, and may, along with increased nutrient inputs, impact fungal assemblages found at these sites.

Finally, marine (seagrass and algal) detritus contained fewer marine ascomycetes than emergent plant detritus or wood as assessed morphologically, and marine detritus was dominated by *Corollospora* species. Bacteria may play a greater role in degrading this substrate type, which was decomposed after 3 months of laboratory incubation during our study.

We observed a change in marine ascomycete community composition with latitude in both Texas and Florida, similar to patterns found in other geographical locations for marine fungi^54–56^, as well as for Ingoldian mitosporic ascomycetes^57,58^, and freshwater ascomycetes^41^. The studies of Hughes^50^ and Booth and Kenkel^55^ both examined larger geographical regions. Through examination of our T-RFLP data, we found that: (i) similar substrates collected at similar latitudes may play a role in shaping intertidal communities, and (ii) the high β diversity observed between Galveston Island in Texas and Caladesi Island in Florida may indicate a role of longitude in structuring these two intertidal fungal communities. Differences in fungal community composition from the southern sites in Texas and Florida may be due to distance and dispersal limitation across the Gulf of Mexico.

Although sampling sites did not vary greatly in salinity nor pH, under predicted increasing ocean acidification regimes, the importance of marine fungi in biogeochemical cycling is expected to increase^59^. Water temperature may play a role in determining marine fungal distributions^24,27^. However, our CCA analysis revealed no effect of either water temperature or salinity on ascomycete species richness or relative abundance from the intertidal substrates we sampled from the U.S. Gulf of Mexico. Our BEST analysis examining environmental variables revealed no significant correlation of water temperature, salinity, pH or season with ascomycete relative species abundance (Rho = 0.234, *P* > 0.1). As sampling occurred before the Deepwater Horizon oil spill (April-2010), our findings contribute pre-oil spill sand beach biodiversity data and marine fungal distribution trends within this economically important oceanographic region. Bik et al.^60^ noted dramatic changes in sediment fungal communities after the Deepwater Horizon oil spill in the U.S. Gulf of Mexico. Post-spill fungal assemblages had low richness and an abundance of hydrocarbon-degrading genera, compared to prior smaller, more diverse fungal assemblages.

In summary, we found: (i) similar sand beach fungal communities at similar latitudes (based on Sørenson’s similarity index & β diversity comparisons among sites, as well as cluster analysis and NMDS of T-RFLP data) and significant ascomycete community differences between 4 site pairs, which may indicate roles of both latitude and longitude in structuring marine ascomycete communities. (ii) Species richness increased with decreasing latitude, perhaps due to increased substrate diversity at the southern collection sites. (iii) Small seasonal differences in fungal species richness and relative abundance were noted for marine plant detritus, and for saltmarsh detritus at Galveston Island, Texas. However, we noted no statistically significant seasonal trends for our study region. Finally, (iv) ANOSIM revealed significantly different fungal communities between the detrital substrate type pairs Marine/Emergent and Marine/Wood.

## Methods

### Collection sites and sampling

Collection sites (*n* = 9) were located along the United States (US) Gulf of Mexico coastline (Fig. 1; Supplementary Table S4). From these sites a total of 750 samples of beach detritus, sand, and seafoam were collected within December 2008 to May 2010. Beach intertidal sites were chosen from Texas and Florida to encompass latitudinal and longitudinal gradients that ensured a diversity of marine substrates, and therefore a diversity of marine fungi, would be documented^61^. As human impacts may decrease diversity and frequency of occurrence of marine fungi in intertidal beach zones^62,63^, sites were chosen on undeveloped beach sections to minimize effects from human disturbance. Besides Texas and Florida sites, West Ship Island (WS in Fig. 1; Mississippi) was also included and allowed additional biogeographic coverage (i.e., combined sites cover the southwest, northwest, north-central, northeast and southeast US Gulf of Mexico). Furthermore, site ‘EB’ for ‘East Beach’, Mississippi, was also sampled weekly (Fig. 1) to assess changes at shorter timescales. Substrates were collected from the intertidal zone at each site (except EB) once in winter (within December 2008–February 2009) and once in summer (within July–September 2009), with 6 months between sampling events at the same site. Sampling occurred every other month at WS (Mississippi) during April 2009–February 2010, and weekly at EB (Mississippi) during May 2010 to investigate changes over shorter timescales. For barrier-island locations (all except BH and EB), intertidal substrates came from the open-water Gulf of Mexico side of each island along a 1 km transect on a falling tide. Replication for each transect was as follows (subject to substrate availability): seafoam [*n* = 20], sand [*n* = 10], marine plant detritus (algae/seagrass) [*n* = 5], emergent plant detritus (saltmarsh/mangrove) [*n* = 5], and driftwood [*n* = 10]. Worm tubes and shells were collected opportunistically, as available. Only solid substrates submerged in seawater for a considerable amount of time were collected (presence of decay or colonization by other marine organisms such as barnacles). Solid substrates (20 g) were transported to the lab on ice, individually stored in sterile plastic zippered bags with a sterile seawater dampened paper towel. Seafoam and sand were collected with a sterile spoon-type skimmer and placed in sterile 50 mL conical tubes and kept on ice. In the lab 250 mg subsamples of abundant substrates were immediately transferred to –20 °C for future DNA extraction. Microscope mounts of unstained seafoam and seafoam stained with lactophenol cotton blue were made immediately upon arrival; remaining seafoam was stored at 4 °C. Environmental data were collected at each site (water temperature, salinity, and pH; see Supplementary Table S5). Contaminant controls during sampling included sterilization of all tools with 95% ethanol between samples, and use of sterile disposable nitrile gloves which were changed between samples. Samples were stored on ice and opening of sterile plastic zippered bags was minimized to reduce exposure to any airborne contaminants.

### Morphological identifications

Mounted seafoam was examined using a NIKON Eclipse 80 microscope with Nomarski interference contrast optics for the presence of fungal spores. Direct seafoam observation can provide a snapshot of the intertidal fungal assemblage^27^. Solid substrates were transferred to sterile plastic boxes containing paper towels, misted twice weekly with artificial seawater [salinity = 35 ppt] and incubated at room temperature [≈24 °C] under natural day/night lighting conditions. All Texas and Florida collections were examined for fungi within one week of collection, and periodically over 3–12 months depending on the length of each substrate’s decay period^64,65^. Mississippi collections (Sites WS and EB) were examined within one week of collection; however, since Mississippi collections occurred more frequently these data were used to assess changes in fungal communities over shorter timescales.

Marine ascomycetes from seafoam, sand and decaying beach detritus were identified morphologically using light microscopy to directly observe fungal reproductive structures. Ascomata were removed from solid substrates with a flame-sterilized needle, squash-mounted in sterile distilled water or lactophenol cotton blue and examined using the aforementioned microscope. Dichotomous and pictorial keys^66,67^ and relevant literature were employed for identifications based on reproductive structures. Photographs of fungal structures were taken using a SPOT Insight camera and measurements (in μm) were made using SPOT 4.1 software. During incubation and microscopy, dissecting needles were sterilized in 95% ethanol and flamed between samples, and samples were incubated in plastic containers sterilized with 95% ethanol before use. Opening of containers was minimized and conducted in a laminar flow hood.

### Community molecular detection via *ITS* T-RFLP

Molecular characterization of fungal communities associated with intertidal drift substrates (wood, marine plant detritus [seagrass & algae], and emergent plant detritus [saltmarsh & mangrove]) used *ITS* Terminal Restriction Fragment Length Polymorphism (T-RFLP) analysis. Community DNA was extracted from 250 mg intertidal solid substrate samples in triplicate using an UltraClean Soil DNA Kit with a vortex adaptor (MoBio). Fungal *ITS* rDNA was PCR amplified using ascomycete-specific primers *ITS* 1-F^68^ and *ITS* 4-A^69^. *ITS* 1-F was labeled on the 5′ end with the fluorescent dye FAM (6-carboxyfluorescein). Cycling parameters were: 3 min at 95 °C, 35 cycles of 1 min at 95 °C, 30 s at 52 °C and 1 min at 72 °C, then lastly 10 min at 72 °C. Negative control reactions using sterile molecular biology grade water in place of DNA and all other reagents are described previously were run for every PCR. PCR products were cleaned using a QIAquick PCR purification kit (Qiagen), followed by restriction digestion using the *HaeIII* restriction enzyme. Restriction digests were as follows: purified amplified DNA at 100 ng (intertidal drift substrate direct source), 1 μl SURE/Cut buffer M (10 ×) and 1 μl *HaeIII* (10U) enzyme (both from Roche Applied Science), and sterile molecular biology grade H_2_O to equal a 10 μL total reaction volume. Digestions were incubated at 37 °C for 3 h then stored at –20 °C. All molecular reactions were set up in a laminar flow bench which was UV-sterilized between procedures.

T-RFLP samples were analyzed at the University of Illinois Urbana-Champaign Core Sequencing Facility using an Applied Biosystems 3730xl DNA Analyzer. Each fluorescently labeled PCR reaction was processed in triplicate and the resulting three chromatograms were overlaid for each sample using GeneMapper Version 3.7 software. Peaks were standardized using the R-based program PAST^68^. Peak height in T-RFLP chromatograms was used as a proxy for the relative abundance of fungal taxa represented by restriction fragments^69^. Peaks were assumed to be artifacts and removed from analysis if they did not contribute more than 1% to the sum of all peak heights in any individual profile and occurred in less than three profiles^69^. Additionally, peaks < 50 bp or > 550 bp were assumed to be primer and uncut *ITS* sequences, respectively, and were excluded from the analysis. The high number of ascomycete species recovered in the T-RFLP analysis includes ascomycete yeasts, which were not inventoried morphologically. T-RFLP data can be used for relative quantification and statistical analysis, although DNA sequence data cannot be definitively inferred directly from the T-RFLP profile^70^. In cases where more than one peak was observed for the same T-RF, the first peak was used to obtain a representative T-RF size as the second peak was most likely the product of an incomplete restriction digest^71^. For additional caveats of ascomycete *ITS* T-RFLP analysis, see Walker and Campbell^22^.

### Statistics

For morphological presence and absence data (scored as 1 and 0, respectively), a site-by-species matrix was constructed. Within this matrix, substrates within sites were column variables and taxa were row variables.

Fungal community differences due to latitude and longitude were observed as differences in fungal species presences and absences on the same substrate types at different locations. Percent species occurrence was calculated as follows: (# collections of a sporulating species ÷ total of samples collected supporting sporulating fungi) × 100. Species richness was standardized to sampling effort by dividing the number of ascomycete species identified by the number of samples collected for each substrate type.

For each substrate type a rarefaction curve (number of species versus number of samples) was plotted for all eight sites pooled to determine the number of samples required to characterize the mycota of each substrate type. Rarefaction trendlines were plotted via the geom_smooth function in ggplot2^72^ and by using local polynomial regression fitting (loess) as the smoothing function, along with the formula y ~ x, a span of 2, and a confidence interval drawn at 0.95 (colored purple). Alpha and beta diversity were calculated for each site/region based on morphological species identifications. Relative frequency of occurrence was calculated for each species. A two-way ANOSIM was performed in PRIMER v6.1.6^73^ to assess the role of substrate and season in structuring fungal community differences.

For T-RFLP data, beta (β) diversity was calculated using the formula proposed by Wilson and Shmida^74^, the most commonly used method for measuring the continuity of species between communities^75^. Fungal similarity among different sites was further calculated using Sørensen’s similarity index^29^: Sørensen’s similarity index = 2*c*/(*a* + *b*); where *a* = total number of species in the first community; *b* = total number of species in the second community; and *c* = number of species both communities have in common.

To perform multivariate statistical analysis on T-RFLP data, these data were converted to a “sample by taxa table” conveying the different samples (T-RFLP profiles) versus the individual taxa (T-RFs), with peak area as species relative abundance values. Species richness (S), Pielou’s evenness (J') and Shannon diversity (H'(log_e_)) were calculated for each site using the DIVERSE module of Plymouth Routines in Multivariate Ecological Research software (PRIMER v.6.1.6, PRIMER-E Ltd, Plymouth, UK)^76^. All statistical tests were performed using PRIMER v6.1.6 software^73^ unless otherwise noted. T-RF relative species abundances were log (x + 1) transformed to compress the scale of comparison prior to calculation of Bray–Curtis similarity matrices^77^. The effect of latitude on fungal community composition was visualized by CLUSTER analysis using the Group Average algorithm to produce the dendrogram. The similarity percentages (SIMPER) routine was applied to decipher percentage contributions from each T-RF (i.e., each species) to the similarity and dissimilarity of each sample in relation to the others. Two-dimensional non-metric multidimensional scaling (NMDS) was used to visualize ascomycete community similarity, with communities with similar relative species abundances placed closer together in ordination space^78,79^. For NMDS plots the analyses were conducted using the vegan package in R^80^, visualized in ggplot^72^, and made use of abundances values that were *log_b* (*x*) + 1 for *x* > 0 transformed (where *b* = base of the logarithm)^30^. A Shepard plot of obtained versus observed ranks was produced to indicate the quality of the NMDS plot (Supplementary Fig. S2). Other figures were also similarly generated in R version 4.0.3^81^ via RStudio Version 1.3.1093^82^ and ggplot2^72^.

Analyses of Similarity (ANOSIM) tests were conducted to assess the roles of latitude, season and substrate in structuring fungal communities. ANOSIM is a multivariate randomization test analogous to ANOVA performed on a similarity matrix and producing a test statistic, R, which assesses the null hypothesis of no among-group differences. R ≅ 0 when there are no significant differences among groups; greater among group differences are indicated as R approaches –1 or 1. The significance of the R statistic is calculated from randomization tests on the similarity matrix^76^. A two-way crossed ANOSIM with replicates was used to assess the role of season (2 levels: winter and summer) across substrate (5 levels: sand, seafoam, marine, emergent, wood) on the data after it was standardized to sampling effort. The BEST procedure^76^ was used to find matches between the among-sample patterns of the T-RF communities, and any patterns from the environmental variables associated with those samples (see Supplementary Table S5), using the Spearman rank coefficient and a Euclidean distance resemblance measure with 999 permutations.

A Canonical Correspondence Analysis (CCA)^72^ was used to test if variation in community composition was explained by water temperature or salinity. In CCA, the ordination axes are linear combinations of the environmental variables. CCA is a direct gradient analysis; the gradient in environmental variables is known a priori and species abundances (or presence/absences) are a response to this gradient. An eigenanalysis algorithm^83^ was used; ordinations are given as site scores. Environmental variables were plotted as correlations with site scores. Canonical analysis has been used for analysis of aquatic mangrove^84^ and Ingoldian mitosporic fungal communities^85^. CCA was performed using the PAST v.2.15 program^68^ for T-RFLP data analysis in R v.2.14.1 software. Maps were generated in R and RStudio using ggplot2^72^, as well as the following additional R packages: mapdata^86^, maps^87^, devtools^88^, usethis^89^, maptools^90^, and ggrepel^91^. Additional citations^92–99^ were also gathered for Supplementary Table S2.

## Supplementary information

Supplementary information.

## Data Availability

Data generated during this work are included herein and/or within online supplementary data. Additional datasets are available on request (contact corresponding author).
