# Supplementary material for: Fungal diversity and community structure from coastal and barrier island beaches in the United States Gulf of Mexico
Source: Sci Rep. 2021 Feb 16;11:3889. doi: 10.1038/s41598-021-81688-5 (PMC7886894; doi:10.1038/s41598-021-81688-5)
Supplement: Supplementary file 1 — Supplementary information. [file 41598_2021_81688_MOESM1_ESM.pdf]

SUPPLEMENTARY INFORMATION for

Fungal diversity and community structure from coastal and barrier island beaches in the United States Gulf of Mexico

Allison K. Walker[1,*] **& Brent M. Robicheau**[2]

[1]Department of Biology, Acadia University, Wolfville, NS, B4P 2R6, Canada

[2]Department of Biology, Dalhousie University, Halifax, NS, B3H 4R2, Canada

***Corresponding author**: allison.walker@acadiau.ca

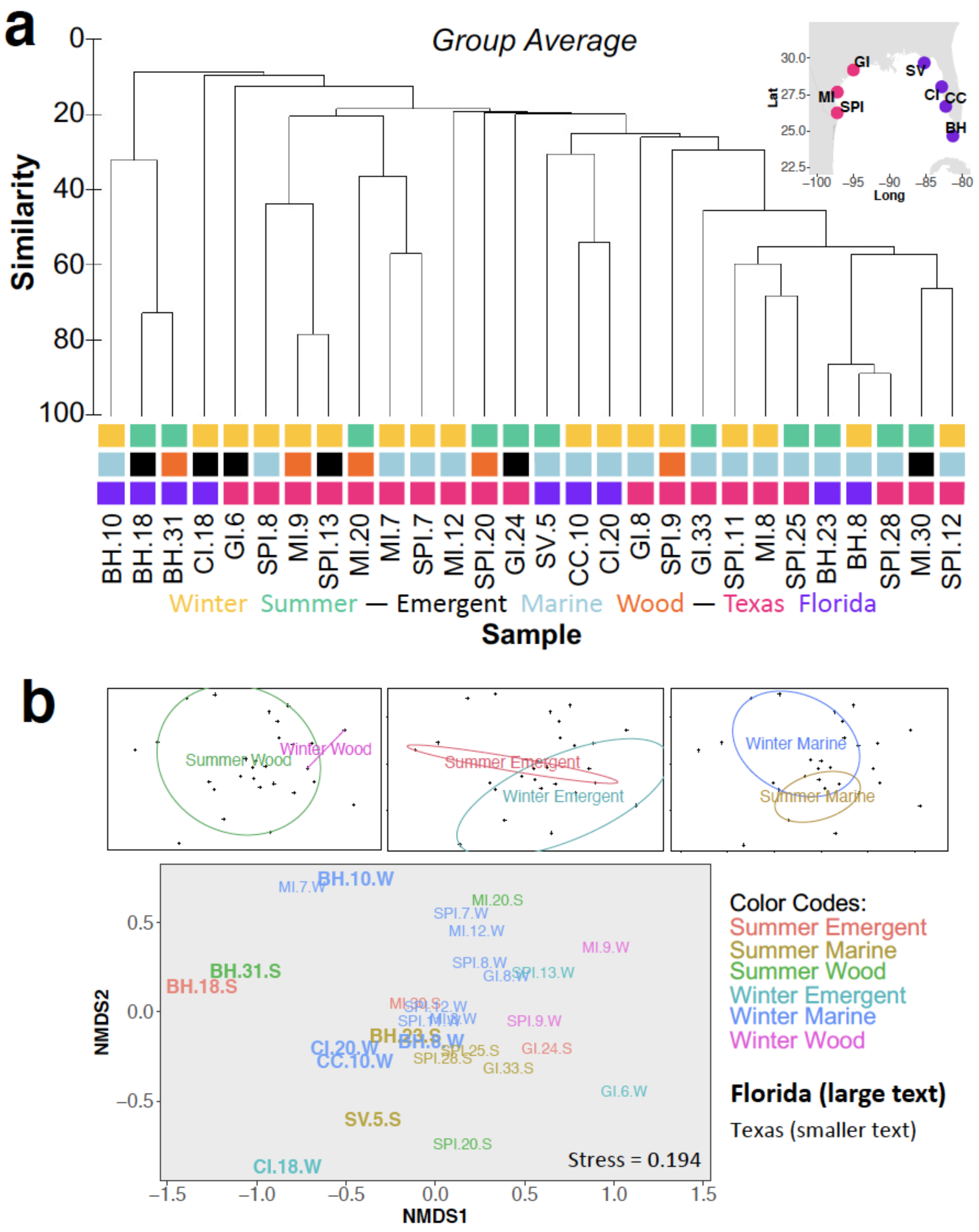

Supplementary Figure S1. U.S. Gulf of Mexico *ITS* T-RF relative species abundance data visualized by (**a**) cluster analysis using the Group Average algorithm, and (**b**) 2D NMDS ordination with samples colored based on season + substrate type. Both methods used a Bray-Curtis similarity matrix with log-transformed data. To help in examining trends in (**b**), smaller graphs with ellipses drawn around all points within unique season + substrate groups are shown. The same axis points were used for smaller graphs. Please refer to Table S4 for full location names.

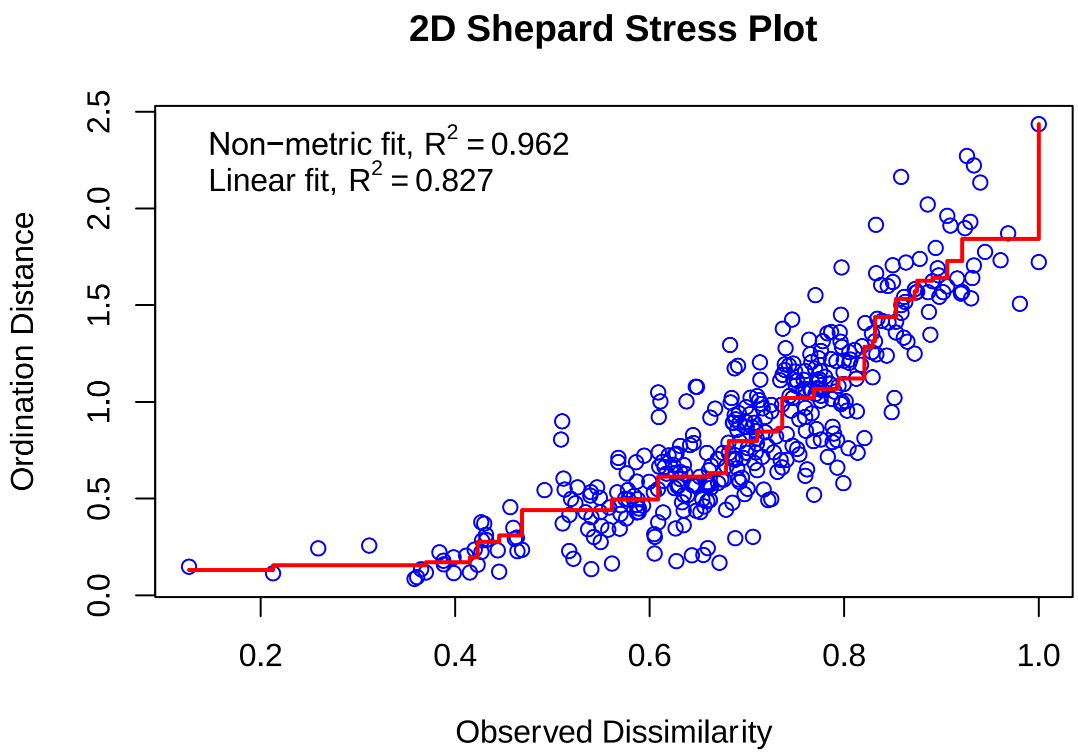

Supplementary Figure S2. Shepard diagram stress plot for NMDS. Stress = 0.194.

Species name and authority
Acrocordiopsis patilii Borse & K.D. Hyde
Anthostomella poecila Kohlm., Volkm.-Kohlm. & O.E. Erikss.
Atrotorquata lineata Kohlm. & Volkm.-Kohlm.
Buergenerula spartinae Kohlm. & R.V. Gessner
Corollospora gracilis Nakagiri & Tokura
Corollospora intermedia E.B.G.Jones
Corollospora maritima Werderm.
Corollospora pulchella Kohlm., I. Schmidt & N.B. Nair
Corollospora ramulosa (Meyers & Kohlm.) E.B.G. Jones & Abdel-Wahab
Haiyanga salina (Meyers) K.L. Pang & E.B.G. Jones
Halobyssothecium obiones (P. Crouan & H. Crouan) Dayarathne, E.B.G. Jones & K.D. Hyde
Haloguignardia decidua Cribb & J.W. Cribb
Halosphaeriopsis mediosetigera (Cribb & J.W. Cribb) T.W. Johnson
Leptosphaeria avicenniae Kohlm. & E. Kohlm.
Lindra sp. 1 I.M. Wilson
Lindra sp. 2 I.M. Wilson
Lindra thalassiae Orpurt, Meyers, Boral & Simms
Loratospora aestuarii Kohlm. & Volkm.-Kohlm.
Lulwoana uniseptata (Nakagiri) Kohlm., Volkm.-Kohlm., J. Campb., Spatafora & Gräfenhan
Massarina ricifera Kohlm., Volkm.-Kohlm. & O.E. Erikss.
Mycosphaerella sp. I Johanson
Mycosphaerella sp. II Johanson
Neomassariosphaeria typhicola (P. Karst.) Y. Zhang ter, J. Fourn. & K.D. Hyde
Periconia sp. Tode
Phaeosphaeria halima (T.W. Johnson) Shoemaker & C.E. Babc.
Phaeosphaeria olivacea Kohlm., Volkm.-Kohlm. & O.E. Erikss.
Phaeosphaeria roemeriani Kohlm., Volkm.-Kohlm. & O.E. Erikss.
Phaeosphaeria spartinicola Leuchtmann
Pleospora pelvetiae G.K. Sutherl.
Sphaerulina orae-maris Linder
Torpedospora radiata Meyers
Varicosporina prolifera Nakagiri

Supplementary Table S1. Species authorities for taxa listed in Figure 3.

Taxon	This Study	Bik et al. (2012)[a]	Thaler et al. (2012)[b]	Al-Nasrawi et al. (2012)[c]	Mata and Cebrián (2013)[d]	Velez et al. (2013)[e]	Velez et al. (2015a)[f]	Velez et al. (2015b)[g]	Simister et al. (2015)[h]	Cassle et al. (2016)[i]	Salamone et al. (2016)[j]
	**Reference**										
Acrocordiopsis patilii	•										
Acarospora		•									
Acremonium		•									•
Acremonium alternatum											•
Alternaria		•		•							•
Alternaria alternata				•							
Anthostomella poecila	•										
Arenariomyces						•	•	•			
Arenariomyces majusculus						•					
Arenariomyces parvulus						•		•			
Arenariomyces trifurcatus						•					
Arenariomyces triseptatus						•		•			
Arthrinium											•
Arthrobotrys sp.					•						
Ascosacculus							•				
Aspergillus		•		•	•				•	•	•
Aspergillus fumigatus										•	
Aspergillus niger				•							
Aspergillus terreus									•		
Atrotorquata lineata	•										
Aureobasidium											•
Aureobasidium pullulans											•
Batrachochytrium dendrobatidis			•								
Barnettozyma californica											•
Bipolaris											•
Buergenerula spartinae	•										
Candida torresii			•								
Ceriosporopsis						•	•				
Ceriosporopsis halima						•					
Cladosporium		•			•						•
Curvularia lunata				•							
Ophiocordyceps sinensis				•							
Corollospora	•					•	•	•			
Corollospora gracilis	•					•		•			
Corollospora intermedia	•										
Corollospora maritima	•					•		•			
Corollospora pulchella	•					•					
Corollospora ramulosa* = *Varicosporium ramulosa	•					•					
Curreya pityophila			•								
Curvularia											•
Paradendryphiella arenariae					•						
Penicillium javanicum				•							
Exophiala											•
Exophiala heteromorpha											•
Exserohilum rostratum					•						

Fusarium sp.				•	•				•		•
Neocosmospora solani				•					•		
Fusarium fujikuroi				•							
Haiyanga salina	•					•					
Halenospora					•		•				
Halobyssothecium obiones	•										
Haloguignardia decidua	•										
Halosphaeriopsis mediosetigera	•										
Halenospora varia					•						
Humicola phialophoroides											•
Trichoderma lixii				•							
Leptosphaeria avicenniae	•										
Phaeosphaeria sp.						•					
Letendraea helminthicola											•
Lignincola laevis						•					
***Lindra* sp.**	•				•	•		•			
Lindra crassa						•					
Lindra thalassiae	•				•	•		•			
Lineolata rhizophorae						•					
Loratospora aestuarii	•										
Lulwoana uniseptata	•										
Lulworthia sp.						•	•	•			
Lulworthia grandispora								•			
Malassezia restricta											•
Massarina ricifera	•										
Mortierella											•
Mucor											•
Mucor moelleri											•
***Mycosphaerella* sp.**	•					•					
Näis inornata						•					
Neomassariosphaeria typhicola	•										
Nigrospora											•
Ochroconis											•
Paecilomyces											•
Penicillium sp.				•	•						•
Pencillium decumbens				•							
***Periconia* sp.**	•										
Pestalotia											•
Pestalotiopsis sp					•						•
Phaeoacremonium											•
Phaeosphaeria halima	•										
Phaeosphaeria olivacea	•										
Phaeosphaeria roemeriani	•										
Phaeosphaeria spartinicola	•										
Phialophora sp.					•						
Phoma sp.					•						•
Phycomyces											•
Pithomyces											•
Stemphylium vesicarium				•							
Pleospora pelvetiae	•										
Pseudeurotium hygrophilum			•								
Pseudozyma											•
Moesziomyces aphidis											•
Purpureocillium lilacinum											•
Remispora							•				
Rhizopus		•									
Rhodocollybia		•									

Buckleyzyma aurantiaca											•
Rhodotorula mucilaginosa											•
Rhopalomyces											•
Savoryella							•				
Scopulariopsis									•		•
Scopulariopsis brevicaulis									•		
Sepedonium											•
Sphaerodes retispora				•							
Sphaerulina orae-maris	•										
Spiromyces											•
Stachylidium											•
Talaromyces											•
Tilletiopsis											•
Torpedospora						•	•				
Torpedospora radiata	•					•					
Humicola alopallonella					•						
Trichoderma sp.					•						•
Trichoderma harzianum											•
Trichophyton											•
Trichosporon											•
Varicosporina prolifera	•										
Williopsis											•

[a] Sediment samples; Dauphin Island and Mobile Bay, Alabama, and Grand Isle, Louisiana USA (Bik et al. 2012).
[b] Deep-sea methane seeps; Alaminos Canyon USA (Thaler et al. 2012).
[c] Sand samples contaminated with oil; Pensacola beach (Al-Nasrawi et al. 2012).
[d] Surface sterilized *Halodule wrightii* and *Thalassia testudinum*; Big Lagoon State Park, Florida, USA (Mata and Cebrián 2013).
[e] Mesobeach sediment; 12 sites along Mexican Gulf of Mexico (Velez et al. 2013).
[f] Intertidal sediment; State of Tabasco shoreline, Mexico (Velez et al. 2015a).
[g] Washed-up detritus, tourist beaches on Cozumel Island, Mexican Caribbean (Valez et al. 2015b).
[h] Oil-soaked sand paddies; Alabama Shores and Dauphin Island USA (Simister et al. 2015).
[i] Deceased dolphin tissue; near Sand Key, Florida, USA (Cassle et al. 2016).
[j] Artifical reef biofilms; Mississippi Sound, USA (Salamone et al. 2016).

Supplementary Table S2. Fungal species reported from the Gulf of Mexico from marine sediment, seawater, seagrass, and detritus since 2012. When only genus is provided, it is because the study only provided genus-level identification; it is not implied that the same ‘unknown’ species was identified across studies.

Species richness (S), Pielou's evenness (J') and Shannon diversity (H'[log $_e$])by site, as calculated from T-RF species richness and relative abundance data

Site	S	J'	H'(log $_e$)
1. South Padre Island	57	0.728985	2.947323
2. Mustang Island	61	0.733071	3.013564
3. Galveston Island	46	0.769617	2.946589
5. St. Vincent Island	7	0.769133	1.496663
6. Caladesi Island	25	0.796515	2.563881
7. Cayo Costa Island	11	0.687413	1.648345
8. Bahia Honda	46	0.72789	2.786829

Supplementary Table S3. Raw data values used in Figure 4.

Site Code	**Location** Coordinates	Development	Sand beaches (km)	Saltmarsh/ Mangrove	SAV/ Algal beds
SPI	**Padre Island** 26°14'52.00" N 97°11'05.10" W	D	112	Neither, but 1,825 km^2 coastal wetlands in nearby Laguna Madre	Neither, but 773 km^2 SAV in nearby Laguna Madre
MI	**Mustang Island** 27°40'14.80" N 97°10'12.30" W	D	29	Neither	SAV only
GI	**Galveston Island** 29°11'20.90" N 94°57'32.10" W	D	51	Neither	Neither
WS	**West Ship Island** 30°12'27.52" N 88°57'49.24" W	UD	9.5	Neither	Neither
SV	**St. Vincent Island** 29°40'36.00" N 85°13'15.30" W	UD	18	Neither	Neither
CI	**Caladesi Island** 28°02'03.80" N 82°49'19.40" W	UD	5	Mangrove only	Neither
CC	**La Costa Island** 26°41'17.90" N 82°15'29.50" W	UD	14.5	Mangrove only	Neither
BH	**Bahia Honda State Park** 24°39'20.50" N 81°16'47.80" W	UD	4	Neither	SAV Only
EB	**East Beach** 30°23'40" N 88°48'36" W	D	4	Saltmarsh	Neither

Supplementary Table S4. Descriptions of intertidal collection sites indicated in Figure 1. Abbreviations: D = developed, UN = undeveloped, SAV = submerged aquatic vegetation.

PHYSICAL-CHEMICAL DATA FOR EACH COLLECTION SITE

Site	Location	Collection Season	GPS Coordinates N	W	Water temperature (C)	Salinity	pH
1	South Padre Island, TX	Winter 08-09	26 14 52.0	97 11 05.1	21.3	31.0	8.26
		Summer2009	26 14 52.0	97 11 05.1	26.6	31.2	8.35
2	Mustang Island State Park, TX	Winter 08-09	27 40 14.8	97 10 12.3	17.0	31.4	8.22
		Summer2009	27 40 14.8	97 10 12.3	28.5	30.1	8.27
3	Galveston Island State Park, TX	Winter 08-09	29 11 20.9	94 57 32.1	19.0	24.0	8.44
		Summer2009	29 11 20.9	94 57 32.1	31.1	29.5	8.28
4	West Ship Island, MS	April 2009	30 12 27.52	88 57 49.24	20.5	21.2	8.20
		June 2009	30 12 27.52	88 57 49.24	34.5	24.0	8.34
		August 2009	30 12 27.52	88 57 49.24	30.2	23.3	8.56
		October 2009	30 12 27.52	88 57 49.24	19.4	19.0	8.47
Site	Location	Collection Season	GPS Coordinates N	W	Water temperature (C)	Salinity	pH
4	West Ship Island, MS	May 2010	30 12 27.52	88 57 49.24	27.4	19.5	9.09
5	St. Vincent NWR, FL	Winter 08-09	29 40 36.0	85 13 15.3	17.6	24.9	8.34
		Summer2009	29 40 36.0	85 13 15.3	30.7	26.6	8.86
6	Caladesi Island State Park, FL	Winter 08-09	28 02 03.8	82 49 19.4	15.4	19.5	8.23
		Summer2009	28 02 03.8	82 49 19.4	30.2	35.0	7.80
7	Cayo Costa State Park, FL	Winter 08-09	26 41 17.9	82 15 29.5	16.0	31.6	8.08
		Summer2009	26 41 17.9	82 15 29.5	29.9	26.6	8.80
8	Bahia Honda State Park, FL	Winter 08-09	24 39 20.5	81 16 47.8	19.0	33.5	8.28
		Summer2009	24 39 20.5	81 16 47.8	29.8	31.1	8.60

Supplementary Table S5. Oceanographic data collected for study sites.
